# The Effect of Fucoidan, a Potential New, Natural, Anti-Neoplastic Agent on Uterine Sarcomas and Carcinosarcoma Cell Lines: ENITEC Collaborative Study

**DOI:** 10.1007/s00005-019-00534-9

**Published:** 2019-01-18

**Authors:** Marcin Bobiński, Karolina Okła, Wiesława Bednarek, Anna Wawruszak, Magdalena Dmoszyńska-Graniczka, Pablo Garcia-Sanz, Iwona Wertel, Jan Kotarski

**Affiliations:** 1 1st Chair and Department of Gynecological Oncology and Gynecology, Medical University of Lublin, Staszica 16, 20-081 Lublin, Poland; 2 Department of Biochemistry and Molecular Biology, Medical University of Lublin, Lublin, Poland; 3 Laboratory of Translational Research, MD Anderson Cancer Center, Madrid, Spain

**Keywords:** Uterine sarcomas, Fucoidan, Targeted treatment

## Abstract

The aim of the study was to assess the activity of fucoidan on the uterine sarcomas (MES-SA and ESS-1) and carcinosarcoma cell lines (SK-UT-1 and SK-UT-1B) and its toxicity on the human skin fibroblasts (HSF). Two uterine sarcomas and two carcinosarcoma cell lines were examined, as a control HSF were used. Cell viability was assessed with MTT test, apoptosis with caspase-3 activity and cell cycle by assessment of DNA synthesis. Fucoidan significantly decreases cell viability in SK-UT-1, SK-UT-1B, and ESS-1 cell lines, such effect was not observed in MES-SA. Fucoidan was not substantially affecting proliferation among normal cells. The tested agent induced apoptosis in all cell cultures used in the experiment. Fucoidan affects cell cycle of all tested cell lines except MES-SA by increasing percentage of cells in G0/sub-G1/G1 phase. Fucoidan do not only affect proliferation but induces apoptosis in selected uterine sarcoma and carcinosarcoma cell lines, so it has potential to be used as cytotoxic agent. Fucoidan seems to be promising anti-cancer agent for endometrial stromal sarcoma and carcinosarcoma.

**Electronic supplementary material:**

The online version of this article (10.1007/s00005-019-00534-9) contains supplementary material, which is available to authorized users.

## Introduction

Uterine sarcomas and carcinosarcoma constitute a rare types of female reproductive organs solid tumors. Sarcomas of the uterus is a heterogeneous group of cancers whose common feature is the histological origin from the mesenchymal tissues. Both sarcomas and carcinosarcoma are characterized by the dynamic development, the capacity to metastasize and postoperative recurrence (Bodner et al. 2003). Among uterine sarcomas we distinguish various histological subtypes including i.a. leiomyosarcoma, endometrial stromal sarcoma. Carcinosarcoma due to its dualistic structure (the presence of both mesenchymal and epithelial component) is nowadays included to the group of carcinoma of the corpus uteri by the majority of authors (Prat 2009). The most common histological type of uterine sarcoma is leiomyosarcoma, which is diagnosed in 1–8.4% cases of malignant uterus cancers (Ramondetta et al. 2016; Ueda et al. 2008).

Rarity and difficulties of preoperative diagnosis of both uterine sarcomas and carcinosarcoma are the main limitations in all the trials aimed to assess the activity of new potential drugs among this type of tumors (Bobiński et al. 2015). Using models of uterine sarcoma e.g. cell lines derived from patients allows to cross this boundary.

Fucoidan is a group of sulfated heteropolysaccharide commonly found in brown seaweeds. Composed primarily of l-fucose residues, and sulfate groups with smaller amounts of d-galactose, d-mannose, d-xylose, d-glucose, uronic acids, and protein (Mak et al. 2014). Recent studies have demonstrated its various biological activities including anti-inflammatory, anticoagulant, anti-HIV and anti-cancer activities (high efficiency in the treatment of a variety of cancers, including breast cancer, prostate cancer, lung cancer, hepatoma and leukemia) (Yang et al. 2013, 2016). Fucoidan was recognized to affect plenty pathways both in neoplastic and non-neoplastic cells. Rui et al. (2017) noticed the ability of fucoidan to inhibit JAK-STAT3 pathway in prostate cancer cell line, furthermore, they revealed that it decreases expression of CD31 and CD105 in tumor tissue. Atashrazm et al. (2016) reported it down regulates AKT and ERK1/2 activation in acute promyelocytic leucemia cells. It is also considered to activate apoptosis and increase intracellular reactive oxygen species in lung cancer (Hsu et al. 2017).

These properties of this substance led to conclusion that it has a chance to be useful in treatment of various types of tumors. Some of pathways listed above were considered to be promising targets in treatment of uterine sarcomas (Cuppens et al. 2015).

This study was aimed to assess the activity of fucoidan on the uterine sarcoma and carcinosarcoma cell lines and its toxicity on the human skin fibroblasts.

## Materials and Methods

### Reagents

Fucoidan isolated from *Undaria pinnatifida* was purchased from Sigma-Aldrich (St. Louis, MO, USA). Roswell Park Memorial Institute 1640 (RPMI-1640), Eagle’s Minimum Essential Medium (MEM), McCoy’s 5a Medium Modified, fetal bovine serum (FBS), trypsin–EDTA were purchased from PAN-Biotech (Aidenbach, Germany), penicillin (100 IU/mL) and streptomycin (100 µg/ml) was obtained from Sigma-Aldrich (St. Louis, MO, USA). PE Active Caspase-3 Apoptosis Kit and Propidium iodide utilizing the PI/RNase Staining Buffer were obtained from Becton Dickinson Biosciences (San Jose, CA, USA).

### Cell Lines and Cultures

Carcinosarcoma cell lines (SK-UT-1, SK-UT1-B), leiomyosarcoma cell line (MES-SA) and endometrioid stromal sarcoma cell line (ESS-1) were obtained from the Laboratorio de Investigación Traslacional (MD Anderson Cancer Center, Madrid). Human skin fibroblasts (HSF) were obtained by the outgrowth technique from skin explants from young volunteers. The cells were cultured in MEM (SK-UT-1, SK-UT1-B), McCoy’s 5a Medium Modified (MES-SA), RPMI-1640 (ESS-1) or DMEM/RPMI (1:1) (HSF) containing 10% (SK-UT-1, SK-UT-1B, MES-SA, HSF) or 20% (ESS-1) FBS and 1% penicillin–streptomycin at 37 °C in a humidified 5% CO_2_ atmosphere. Cells from the 4th to 9th passage were used for all experiments.

### Cell Viability Assay

SK-UT-1, SK-UT-1B, MES-SA, ESS-1 (3 × 10^4^ cells/ml) and HSF (1 × 10^5^ cells/ml) cells were platted on 96-well microplates. The cells of all the lines were incubated in the presence of fucoidan (0.01–5 mg/ml) for 96 h. After that, the cells were incubated for 3 h with the MTT [3-(4,5-dimethylthiazol-2-yl)-2,5-diphenyltetrazolium bromide] solution (5 mg/ml, Sigma, USA). During the time MTT was metabolized by living cells to purple formazan crystals, which were later solubilized in SDS buffer (10% SDS in 0.01 N HCl) overnight. Separate experiments were performed in triplicate. The optical density of the product was measured at 570 nm with the use of an ELX-800 plate reader (Bio-Tek, Instruments, USA) and analyzed using Gen5 software.

### Assessment of Apoptosis

Examined cell lines (SK-UT-1, SK-UT-1B, MES-SA, ESS-1) were placed on 6-well plates (Nunc, Denmark) at a density of 1 × 10^5^/ml and then treated with fucoidan (0.05–5 mg/ml) for 48 h. After that, cells were harvested and washed twice with phosphate-buffered saline. Next, cells were fixed and permeabilized using the Cytofix/Cytoperm Solution according to the manufacturer’s instructions of PE Active Caspase-3 Apoptosis Kit. Finally, cells were washed twice in the Perm/Wash Buffer prior to intracellular staining with PE-conjugated anti-active caspase-3 monoclonal rabbit antibodies. Labeled cells were analyzed by flow cytometer FACSCalibur (Becton Dickinson, San Jose, CA, USA), operating with CellQuest software to quantitatively assess the caspase-3 activity.

### Cell Cycle Analysis

Experiments were performed using the FACSCalibur flow cytometer. Cancer cell lines (SK-UT-1, SK-UT-1B, MES-SA, ESS-1) were treated with different concentrations of fucoidan (0.05–5 mg/ml) for 48 h and then fixed in ice-cold 80% ethanol at − 20 °C for 24 h. After fixation, the cells were stained with propidium iodide utilizing the PI/RNase Staining Buffer (BD Biosciences, USA) according to the manufacturer’s instructions. Acquisition rate was at least 60 events per second in low acquisition mode and at least 10,000 events were measured. Cell cycle analysis was performed using flow cytometry analyzing software Cylchred Version 1.0.2 for Windows (source: University of Wales) and WinMDI 2.9 for Windows (source: facs.scripps.edu/software.html). The cells were acquired and gated using dot plot FL-2 width (*X*) versus FL-2 area (*Y*)-gate to exclude aggregates and analyzed in histograms displaying fluorescence 2-area (yellow-orange fluorescence: 585 nm).

### Statistical Analysis

Significant differences were evaluated using GraphPad Prism 5.0 (GraphPad Software Inc., CA, USA); (one-way ANOVA; Tukey’s post-hoc testing). *p* < 0.05 was considered to indicate a statistically significant difference. Results were presented as mean ± standard deviation (± SD) of the mean.

## Results

### Antiproliferative Activity

**Fig. 1:**
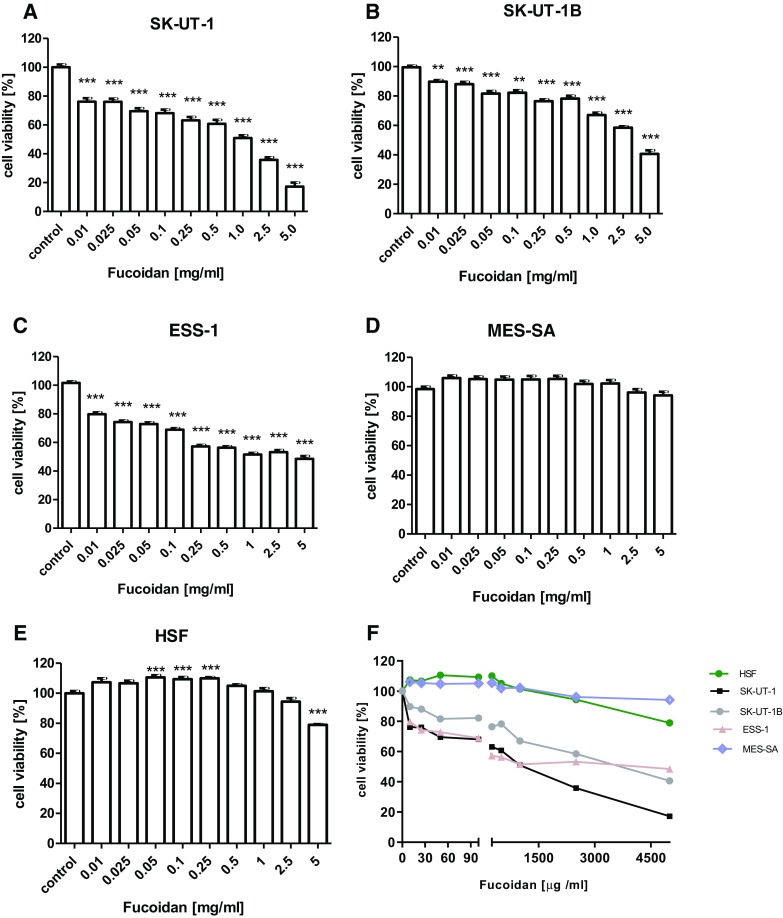
The influence of fucoidan on the proliferation of SK-UT-1 (**a**), SK-UT-1B (**b**), ESS-1 (**c**), MES-SA (**d**) cell lines, human skin fibroblast (HSF) (**e**) and combined chart (**f**). The cells were treated with the fucoidan at various concentrations for 96 h (**p < 0.01, ***p < 0.001 versus the control, one-way ANOVA test)

Our study confirmed the antiproliferative effect of fucoidan in a dose-dependent manner. The antiproliferative effect of fucoidan was investigated in SK-UT-1, SK-UT-1B, ESS-1, MES-SA and HSF using MTT cell viability assay. The results showed that fucoidan significantly decreases cell viability in SK-UT-1, SK-UT-1B, and ESS-1 cell cultures. At the highest concentration (5 mg/ml), the agent reduced cell proliferation to 17.22, 40.68 and 48.51% of control cells after 96 h, respectively. IC50 was 0.966, 3.348 and 0.848 mg/ml, accordingly. In contrast, human sarcoma cell line MES-SA was resistant to fucoidan treatment. Interestingly, fucoidan was not substantially affecting proliferation among normal cells (HSF) in studied range of concentrations (see Fig. 1).

### Proapoptotic Activity

**Fig. 2:**
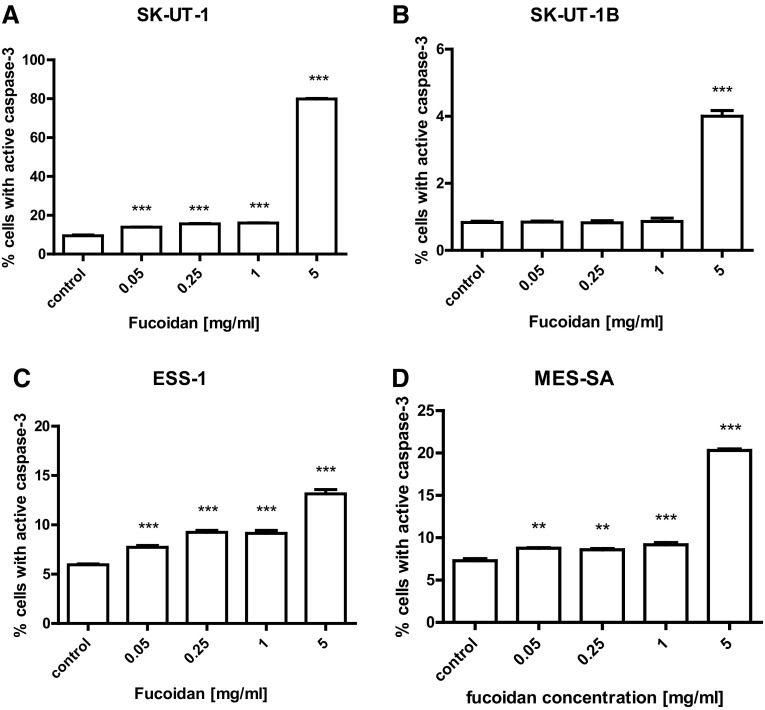
Effects of fucoidan on caspase-3 activation in SK-UT-1 (**a**), SK-UT-1B (**b**), ESS-1 (**c**), MES-SA (**d**) cell lines. Induction of apoptosis by fucoidan after 48 h exposure. Data were analyzed by flow cytometry and results are expressed as mean ± SD of three separate experiments (**p < 0.01, ***p < 0.001 versus the control, one-way ANOVA test)

The treatment of all examined human uterine sarcoma and carcinosarcoma cell lines with fucoidan for 48 h resulted in dose-dependent significant increase in number of apoptotic cells in relation to control. The antiapoptotic effect of agents was determined by the percentage of the cells with active caspase-3 using flow cytometer. Treatment of ESS-1 and MES-SA with 5 mg/ml fucoidan induced apoptosis in 13.5% and 20.31%, respectively. The induction of apoptosis was even more evident in SK-UT-1 cell line with the same fucoidan dose (79.88% apoptotic cells) in contrast to SK-UT-1B (4% apoptotic cells) (see Fig. 2). Representative dot plots are presented on Supplementary Fig. 1.

### Effects of Fucoidan on Cell Cycle Arrest

**Fig. 3:**
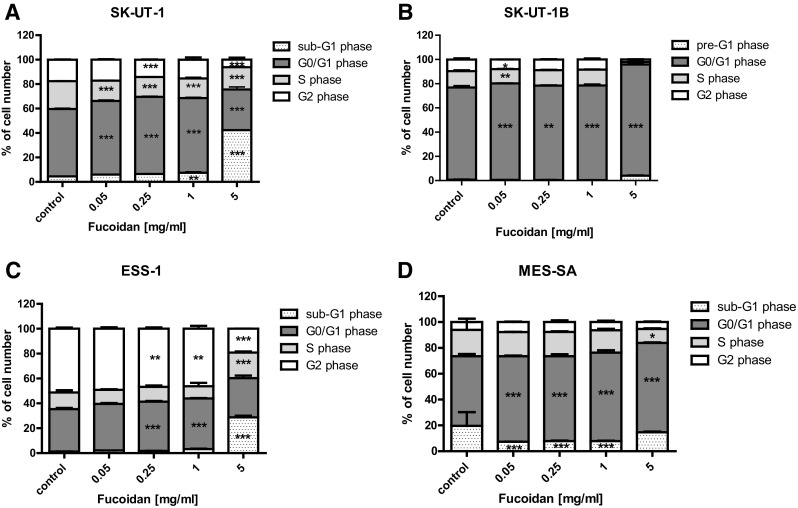
Effect of fucoidan on cell cycle progression in SK-UT-1 (**a**), SK-UT-1B (**b**), ESS-1 (**c**) and MES-SA (**d**) cell lines. The cell lines were incubated for 48 h with fucoidan (0.05–5 mg/ml) and analyzed by flow cytometry. The results are presented as mean ± SD from three separate experiments. Data were analyzed by flow cytometry and results are expressed as mean ± SD of three separate experiments (*n* = 6 per each concentration; *p < 0.05, **p < 0.01, ***p < 0.001 versus the control, one-way ANOVA test)

According to the cell cycle progression analysis we observed, that cell cycle changes induced by fucoidan treatment depended on the human uterine sarcoma cell line used. FACS analysis of PI-stained cells indicated that incubation SK-UT-1 and ESS-1 cells with fucoidan for 48 h led to accumulation of apoptotic cancer cells (sub-G1) corresponding with the cell reduction in the G2 phase. Incubation of the SK-UT-1B cells with the highest concentration of fucoidan resulted in increase of cells number in sub-G1 and G0/G1 phases, followed by the reduction of cell number in G2 phase. The decreased of cell number in G2 phase in MES-SA cell line after fucoidan treatment was not apparent. However, agent induced S phase arrest in MES-SA cell at relatively high concentration. The detailed results of cell cycle analysis are presented on Fig. 3. Representative histograms are presented on Supplementary Fig. 2.

## Discussion

The survey of literature led to consideration that this paper is probably the first one reporting the activity of fucoidan against uterine sarcomas and carcinosarcoma cell lines as well as the first regarding its potential usefulness among these types of cancer in general.

Although the progress in systemic treatment of cancers is undoubtedly, the results of treatment of uterine sarcomas as well as carcinosarcomas still remain unsatisfying (Bobiński et al. 2016). Except form synthesis of new molecules with antineoplastic potential, natural substances are the scope for searching new targeting agents.

As it was mentioned above, fucoidan was found to interact with many pathways in cancer cells. Due to such characteristics it is difficult to indicate particular mechanism of its activity. It was proved that it interferes with genes expression (Rui et al. 2017), epigenetic processes as well as protein expression and its functions (Hsu et al. 2013; Lin et al. 2017). Our understanding of this processes and the relations between them, still remains incomplete. On the other hand, multitarget activity of fucoidan suggests that it is promising agent with ability to overcome tumor resistance induced by activation of evading pathways.

Our experiments showed that fucoidan decreases cell viability among all tested cell lines except MES-SA, while do not substantially affect viability in HSF. The fact that cells viability in normal, human cell line was not decreased significantly by this substance in concentrations below 5 mg/ml allows to expect that it would be safe drug with limited adverse events. Concentration of 5 mg/ml is very high and probably difficult to achieve in human or animal plasma. This concentration was used in the study to asses safe range for normal cell lines. All of conclusions were based on results that were obtained with lower concentrations. In this place, it is worth to note that tolerance and biodistribution of fucoidan is actually under assessment in phase 1 clinical trial (ClinicalTrials.gov Identifier: NCT03422055).

Modern chemotherapy is mostly based on cytostatic agents, that are active against the cells with ability to division, this strategy lead to spectacular response in monoclonal cancers. However, the majority of solid tumors (including uterine sarcomas and carcinosarcoma) is polyclonal with strongly expressed intratumoral heterogeneity, with significant differences between particular cells population in the tumor (Holzmann et al. 2017). Pro-apoptotic activity of fucoidan was previously reported in i.a. colon carcinoma and breast cancer cells (Chen et al. 2014; Hyun et al. 2009; Xue et al. 2017). Our results confirmed such observations among uterine sarcomas cell lines, furthermore we noted that it influence cell cycle by arresting it in G0/G1 (not dividing cells) and sub-G1 (apoptotic cells). In cultures treated with fucoidan we observed increased percentage of cells that were not prone to divide in all cell lines except MES-SA, where low significance was observed only in highest concentration of fucoidan.

The resistance of MES-SA for fucoidan treatment cannot be easily understood because fucoidan’s activity against cancer cells is still not fully described. Several papers revealed that fucoidan affects estrogen receptor pathway so it could be one possible explanations of MESSA resistance (Zhang et al. 2016). Gosland et al. (1993) investigated the impact of estrogen stimulation for chemo-resistance effect in various cell lines. In this study they revealed lack of response in MES-SA line. This reports suggest that the role of this pathway is limited in this cell line, what could partially explains results obtained in our study.

However, we observed that fucoidan induces apoptosis among them. So even if the part of cells population entered the process of apoptosis the other was dividing rapidly and no effect in cell viability was noted. Zhang et al. (2013) reported synergistic effect of fucoidan and cytostatic drugs including cisplatin, tamoxifen and paclitaxel in breast cancer cell lines. Such effect may be caused by fucoidan’s cytostatic activity mentioned above. Consequently, we can expect that its combination with cytostatic drugs, which are expected to interrupt cells divisions may result with synergic effect, but these expectations require further investigations.

The fact of dualistic nature of carcinosarcomas (Chen et al. 2017) combined with revealed in this research fucoidan influence on cell viability, apoptosis as well as cell cycle arrest in carcinosarcoma cell lines may suggest fucoidan’s potential activity against endometrial cancer cells.

Taking into consideration the results obtained in this study we can state that fucoidan express not only cytostatic activity but cytotoxic as well (by inducing apoptosis).

The results of our study justify further research on fucoidan in uterine sarcomas. Experiments on 3D cultures and patients derived xenografts are necessary to confirm its activity in vivo and to assess the possibility to achieve concentrations affecting tumor cells in living organisms.

In conclusion, fucoidan do not only affect proliferation but induces apoptosis in uterine sarcoma and carcinosarcoma cell lines, so it has potential to be used as cytotoxic agent. In vitro experiment suggest that fucoidan seems to be promising anti-cancer agent for uterine sarcomas.

## Electronic supplementary material

Below is the link to the electronic supplementary material.

Supplementary material 1 Supplementary Fig. 1 Detection of apoptotic cells. Induction of apoptosis by fucoidan. SK-UT-1B cell lines was incubated for 48 h with different fucoidan concentration (0.05–5 mg/ml) and analyzed by flow cytometry. Symbol R1 indicates the number of all cells (left), R2: cells with active caspase-3. **A**: control, **B**: 0.05 mg/ml, **C**: 0.25 mg/ml, **D**: 1 mg/ml, **E**: 5 mg/ml) (PPT 914 KB)

Supplementary material 2 Supplementary Fig. 2 Effect of fucoidan on the cell cycle progression in SK-UT-1B. Flow cytometry histograms, (**A**) control, (**B**) 0.05 mg/ml, (**C**) 0.25 mg/ml, (**D**) 1 mg/ml, (**E**) 5 mg/ml. M4 gate (sub-G1 phase), M1 gate (G0/G1 phase), M2 gate (S phase), M3 gate (G2 phase) (PPT 848 KB)
