# Supplementary figures and images for: The Effect of Fucoidan, a Potential New, Natural, Anti-Neoplastic Agent on Uterine Sarcomas and Carcinosarcoma Cell Lines: ENITEC Collaborative Study
Source: Arch Immunol Ther Exp (Warsz). 2019 Jan 18;67(2):125–31. doi: 10.1007/s00005-019-00534-9 (PMC6420609; doi:10.1007/s00005-019-00534-9)

## Slide 1
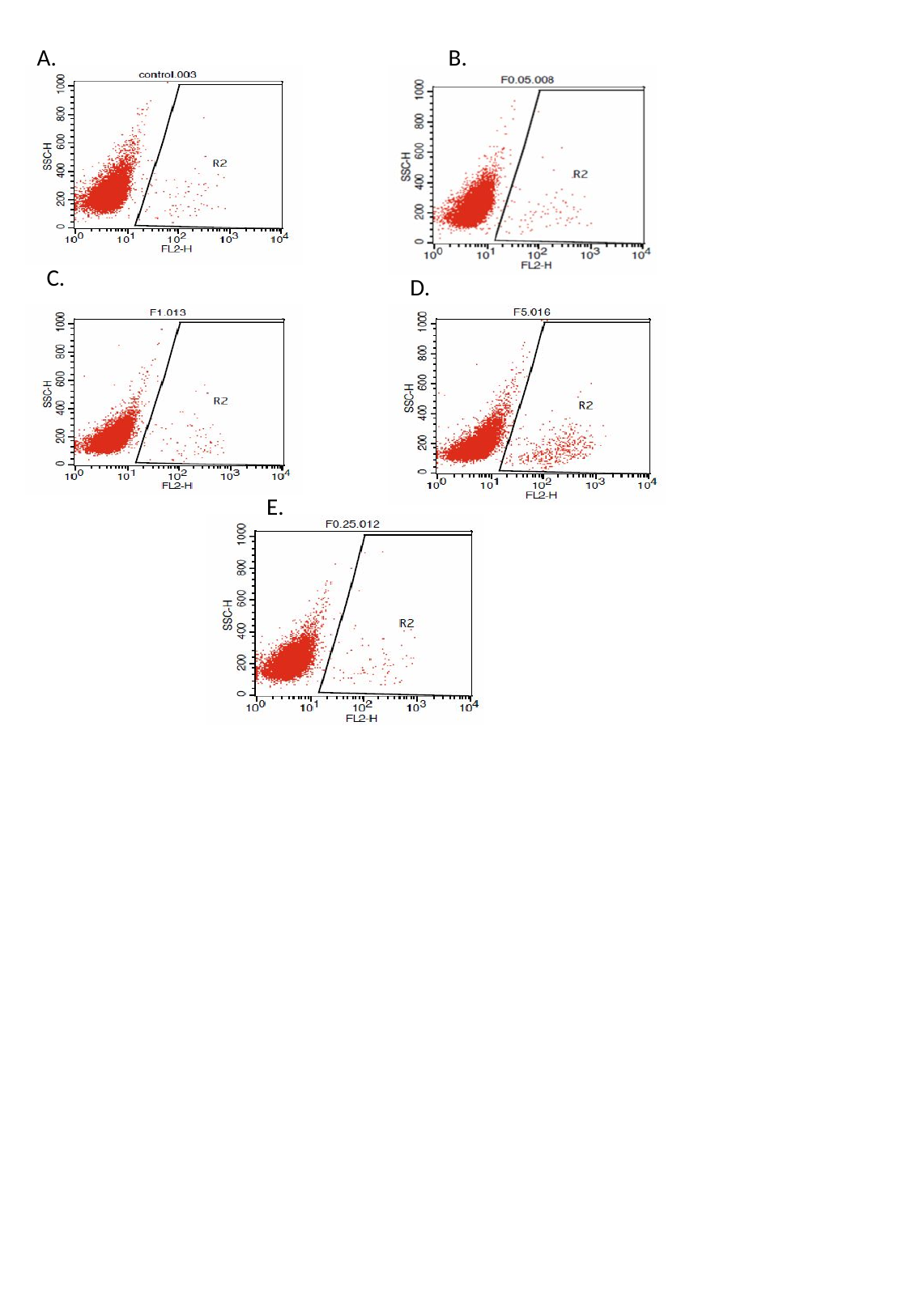

A.
B.
C.
D.
E.

Supplement: Supplementary file 1 — Supplementary material 1 Supplementary Fig. 1 Detection of apoptotic cells. Induction of apoptosis by fucoidan. SK-UT-1B cell lines was incubated for 48 h with different fucoidan concentration (0.05–5 mg/ml) and analyzed by flow cytometry. Symbol R1 indicates the number of all cells (left), R2: cells with active caspase-3. A: control, B: 0.05 mg/ml, C: 0.25 mg/ml, D: 1 mg/ml, E: 5 mg/ml) (PPT 914 KB) [file 5_2019_534_MOESM1_ESM.ppt]

## Slide 1
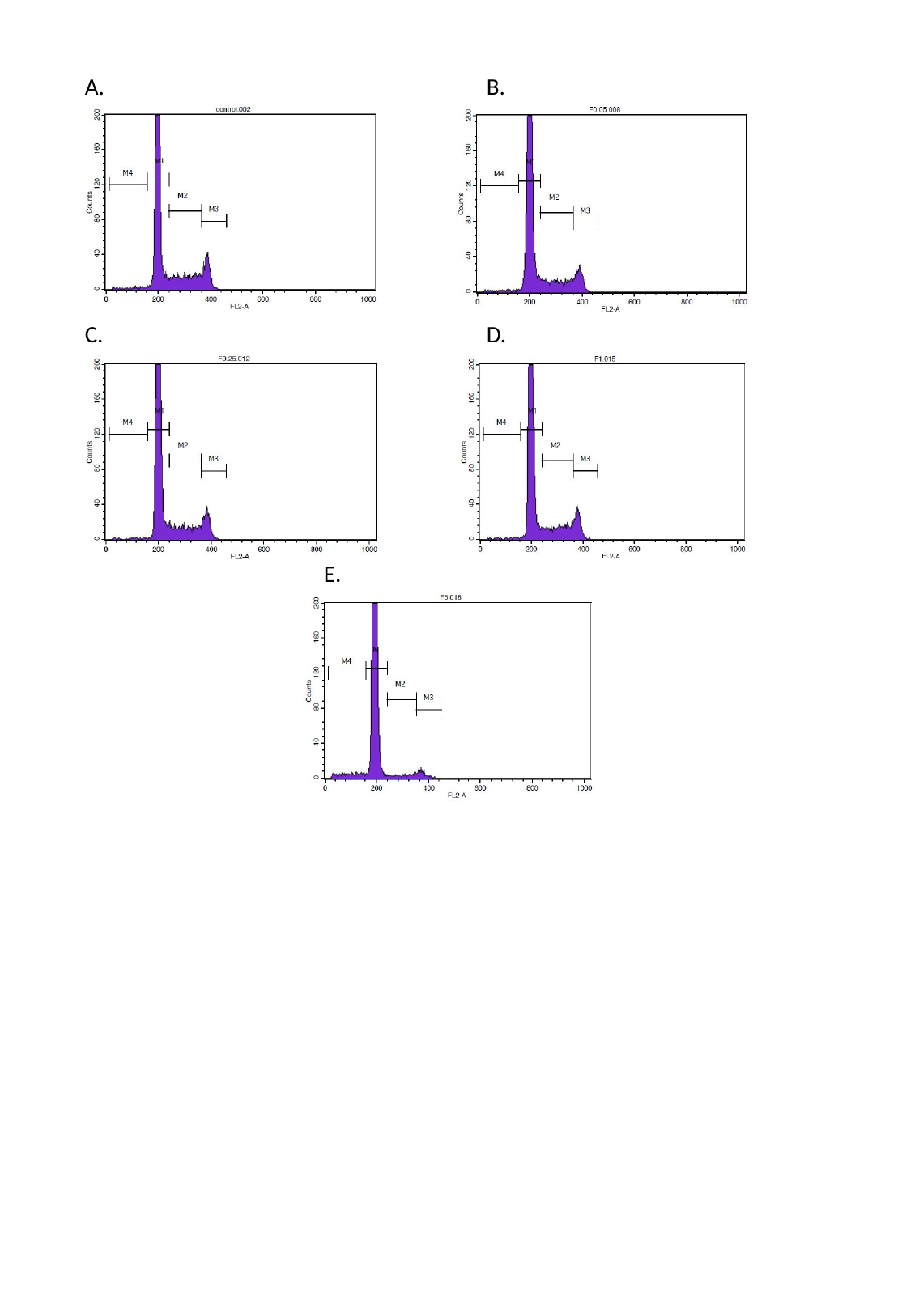

A.
B.
C.
D.
E.

Supplement: Supplementary file 2 — Supplementary material 2 Supplementary Fig. 2 Effect of fucoidan on the cell cycle progression in SK-UT-1B. Flow cytometry histograms, (A) control, (B) 0.05 mg/ml, (C) 0.25 mg/ml, (D) 1 mg/ml, (E) 5 mg/ml. M4 gate (sub-G1 phase), M1 gate (G0/G1 phase), M2 gate (S phase), M3 gate (G2 phase) (PPT 848 KB) [file 5_2019_534_MOESM2_ESM.ppt]
